# Serotype Profile of Nasopharyngeal Isolates of *Streptococcus pneumoniae* Obtained from Children in Burkina Faso before and after Mass Administration of Azithromycin

**DOI:** 10.4269/ajtmh.19-0944

**Published:** 2020-06-08

**Authors:** Soumeya Hema-Ouangraoua, Issaka Zongo, Nongodo Firmin Kabore, Nikiema Frédéric, Rakiswende Serge Yerbanga, Halidou Tinto, Yves Daniel Compaore, Irene Kuepfer, Daniel Chandramohan, Brian Greenwood, Jean Bosco Ouedraogo

**Affiliations:** 1 Centre MURAZ, Bobo-Dioulasso, Burkina Faso;; 2 Institut de Recherche en Sciences de la Santé (IRSS), Bobo-Dioulasso, Burkina Faso;; 3 London School of Hygiene & Tropical Medicine, London, United Kingdom

## Abstract

Mass drug administration (MDA) with azithromycin (AZ) has been used successfully to control trachoma. However, several studies have shown that MDA with AZ has led to the emergence of resistance to AZ in *Streptococcus pneumoniae.* The emergence of resistance to AZ has also been observed when this antibiotic was combined with the antimalarials used for seasonal malaria chemoprevention (SMC). The development of antibiotic resistance, including resistance to AZ, is sometimes associated with the emergence of a bacterial clone that belongs to a specific serotype. We hypothesize that the increase in resistance of *S. pneumoniae* observed after 3 years of SMC with AZ might be associated with a change in the distribution of pneumococcal serotypes. Therefore, 698 randomly selected isolates from among the 1,468 isolates of *S. pneumoniae* obtained during carriage studies undertaken during an SMC plus AZ trial were serotyped. A polymerase chain reaction (PCR) multiplex assay using an algorithm adapted to the detection of the pneumococcal serotypes most prevalent in African countries was used for initial serotyping, and the Quellung technique was used to complement the PCR technique when necessary. Fifty-six serotypes were detected among the 698 isolates of *S. pneumoniae*. A swift appearance and disappearance of many serotypes was observed, but some serotypes including 6A, 19F, 19A, 23F, and 35B were persistent. The distribution of serotypes between isolates obtained from children who had received AZ or placebo was similar. An increase in AZ resistance was seen in several serotypes following exposure to AZ. Mass drug administration with AZ led to the emergence of resistance in pneumococci of several different serotypes and did not appear to be linked to the emergence of a single serotype.

## INTRODUCTION

Many sub-Saharan African countries use azithromycin (AZ) in mass drug administration (MDA) programs to control trachoma.^1^ Previous studies have shown that in communities with a high child mortality, MDA with this macrolide can significantly reduce mortality in children younger than 5 years.^2–6^ Several studies of MDA with AZ for the control of trachoma have noted a temporary increase in the resistance of *Streptococcus pneumoniae* to AZ.^3,4,6–14^ In a study conducted in Burkina Faso, in which the impact of the addition of AZ to the antimalarials used for seasonal malaria chemoprevention (SMC) on child mortality and morbidity was investigated, we found that the prevalence of pneumococcal carriage decreased over time but that there was an increase in the prevalence of *S. pneumoniae* to AZ which persisted for a year after AZ administration has been stopped. Resistance to AZ was more pronounced in the children receiving AZ than in the controls.^15^

The emergence of antibiotic resistance, including resistance to AZ, in *S. pneumoniae* is sometimes linked to the emergence of a single serotype.^16,17^ Thus, we considered that we might find changes in the serotype distribution of nasopharyngeal isolates of *S. pneumoniae* before and after administration of AZ with SMC for 3 years. This article reports the findings of a study designed to test this hypothesis.

## MATERIALS AND METHODS

### Study design.

Samples for this study were collected during the course of a large clinical trial which investigated the potential benefit of adding AZ to SMC with sulfadoxine–pyrimethamine plus amodiaquine (SP + AQ) on child mortality and admissions to hospital which was conducted from 2014 to 2016 in children aged 3–59 months in Burkina Faso and Mali. No impact was observed on the primary trial end point of death or hospital admission, but a reduction in clinic attendances with acute respiratory or gastrointestinal infections and skin diseases accompanied by fever was observed.

During each malaria transmission season, children enrolled in the trial received four courses of SMC per year 1 month apart. Infants aged 3–11 months received SP 250 mg/12.5 mg and AQ 75 mg on day 1 and AQ 75 mg on days 2 and 3. In addition, they received AZ 100 mg or matching AZ placebo on days 1, 2, and 3. Children aged 1–4 years received double these doses. Sulfadoxine–pyrimethamine + amodiaquine was supplied by Guilin Pharmaceutical (Shanghai, China), and AZ and matching placebo by CIPLA (Mumbai, India). All doses of treatments were given by trial staff. Coverage with monthly treatments was high, with more than 80% of children receiving three or four rounds of treatment each year.^6^

In 2013, Burkina Faso began the introduction of pneumococcal conjugate vaccination 13 (PCV13) into the Expanded Programme of Immunization. Coverage with three doses of PCV13 was 58.3% before the 2015 malaria transmission season. An increase in the coverage rate was observed in 2016.

A sub-study to determine the impact of administration of AZ in association with SMC on the resistance of *S. pneumoniae* to this antibiotic was nested within the main SMC + AZ trial. Each year, over a period of 3 years, 400 Burkinabe children were randomly selected for inclusion in a nasopharyngeal carriage study. Different children were selected each year. After written, informed consent had been obtained from a parent or guardian, a nasopharyngeal swab was collected before the first round of SMC and 1 month after the last round of SMC each year. The trial was approved by the National Ethics Committee in Burkina Faso (approval number 2016-11-126) and by the Ethics Committees at the London School of Hygiene & Tropical Medicine. It was also registered on the website, clinicaltrials.gov (NCT02211729).

### Collection of nasopharyngeal samples.

Nasopharyngeal swabs were collected using a calcium alginate swab from the posterior wall of the nasopharynx and immediately transferred to vials containing skim milk–tryptone–glucose–glycerol medium. Vials were stored in a cold box before transfer to the laboratory within 8 hours of collection, and the vials were stored at −80°C in accordance with the WHO protocol for the evaluation of pneumococcal carriage.

### Laboratory testing.

Details about the isolation and characterization of pneumococci have been presented previously.^15^ A single representative colony, selected on the basis of its appearance, was sampled per plate. Antibiotic sensitivity was measured using antibiotic impregnated discs. Resistance to penicillin and macrolides was confirmed by E-test strips. Serotyping was performed by sequential multiplex PCR using an algorithm designed for Africa.^18^ Eight series of PCRs including four to five serotypes per series were performed for the detection of 40 pneumococcal serotypes. DNA extracts were obtained by suspending frozen isolates in 500 μL PBS buffer and heating them to 100°C for 10 minutes (see Supplemental). All pneumococcal isolates determined to be non-typeable by PCR or for which the serotype was unclear were further tested by the Quellung reaction with antisera prepared in the Streptococcus Laboratory at the CDC, Atlanta.

### Data management and statistical analysis.

For logistic reasons, it was not possible to serotype all the pneumococcal isolates obtained during the 3 years of the study. A random selection of approximately half the isolates (698/1,418) (49.2%) was selected for serotyping.

Baseline sociodemographic and clinical data were collected on hard copy case report forms, and laboratory results were recorded in the first instance in laboratory books before being transferred into an electronic database using Excel. Data were cleaned and analyzed with Stata version 15.0 software (Stata Corp LLC, College station, TX). Data were presented as proportions and compared using chi^2^ or Fisher’s exact test. A Poisson regression model was used to estimate the prevalence ratios between treatment arms at each survey. Vaccine serotypes (0 or 1) and resistance to azithromycin (0 or 1) were the dependent variables, whereas the treatment arm (0 or 1) and the survey (1–6) were the independent ones. The significance threshold used for statistical tests was *P* < 0.05.

## RESULTS

**Figure 1.**
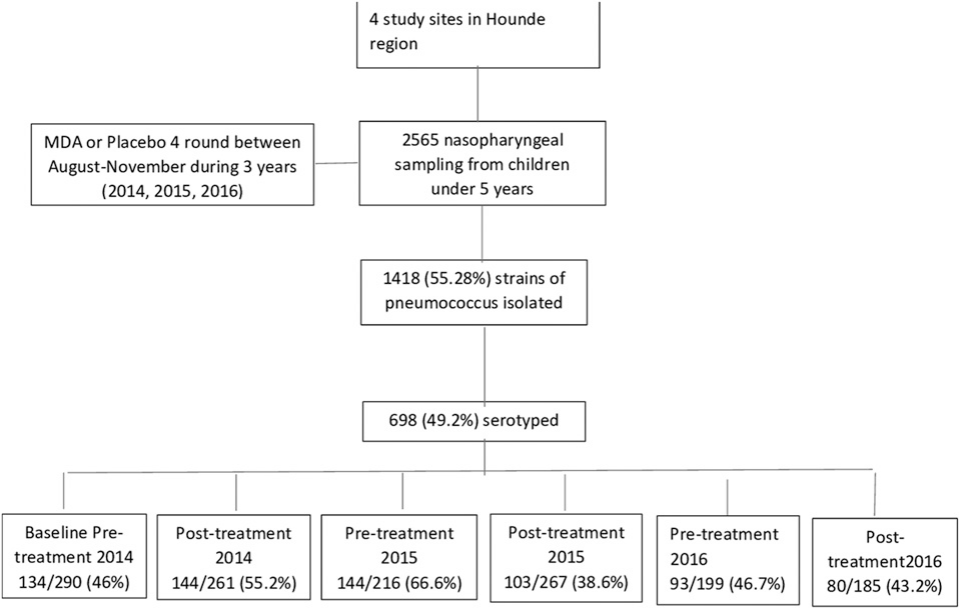
Flowchart for the study of the effect of azithromycin administration on nasopharyngeal serotype of *Streptococcus pneumoniae*, Burkina Faso, 2014–2016. Numbers in the lowest row of the boxes show the numbers of isolates that were serotyped at each survey.

During the course of six carriage surveys undertaken between 2014 and 2016, 2,565 nasopharyngeal samples were taken from children aged 3–59 months, from which 1,418 isolates of *S. pneumoniae* were obtained. Six hundred ninety-eight of these isolates, equitably distributed among the surveys, were randomly selected for serotyping (Figure 1). The PCR and/or Quellung test detected 56 different serotypes.

### Baseline survey.

**Figure 2.**
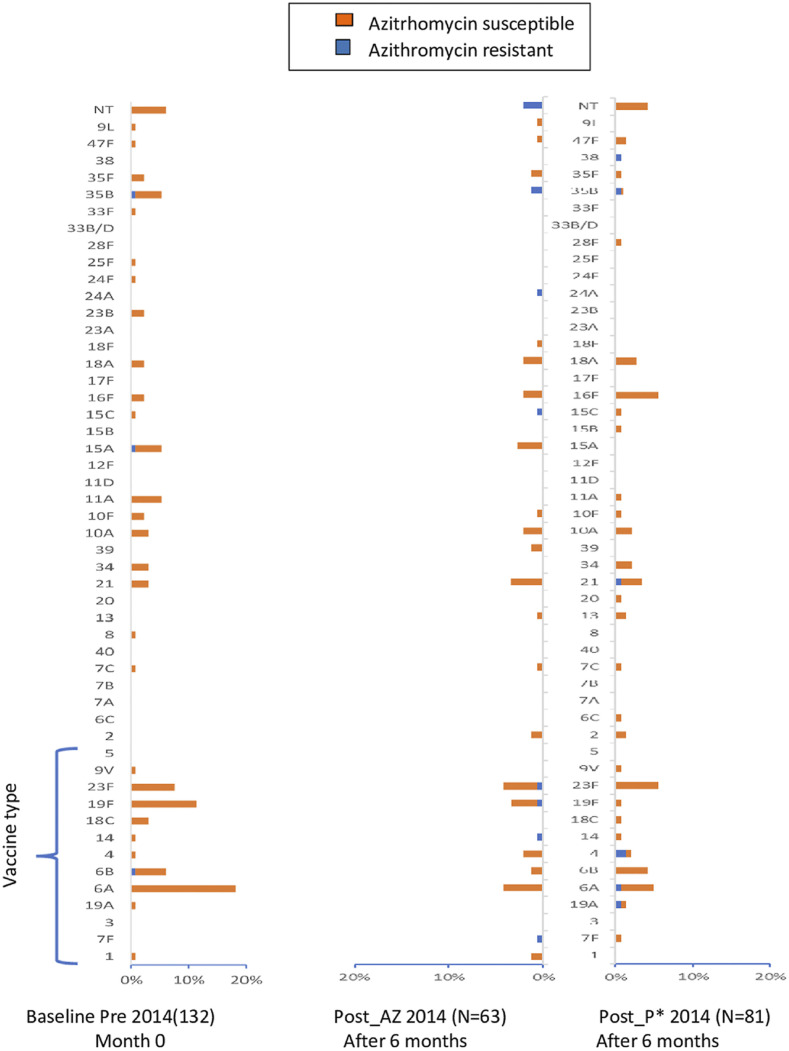
Distribution of nasopharyngeal pneumococcal serotypes before and after azithromycin administration in 2014. The proportions of serotypes detected at the respective study visit are shown: orange bars represent azithromycin-susceptible isolates, and blue bars represent azithromycin-resistant isolates. Pneumococcal conjugate vaccination 13 vaccine-type (VT) serotypes are grouped at the bottom of the figure. This figure appears in color at www.ajtmh.org.

*Streptococcus pneumoniae* was isolated from 230 (67%) of the 430 nasopharyngeal samples collected at the first pre-intervention survey (baseline), and 134 (58.3%) of these isolates were serotyped. Thirty-two unique serotypes were identified and classified into vaccine-type (VT) and non–vaccine-type (NVT) serotypes, as shown in Figure 2. Vaccine serotypes accounted for 49.3% of the isolated serotypes; 37.7% of children who carried a pneumococcus of VT had received at least one dose of PCV13.

The most frequently encountered serotypes were 6A, 19F, 23F, and 6B, which represented 42.2% of the serotyped isolates. A high prevalence of serotype 6A (17.8%) was isolated among the strains serotyped.

### Subsequent pretreatment surveys.

**Figure 3.**
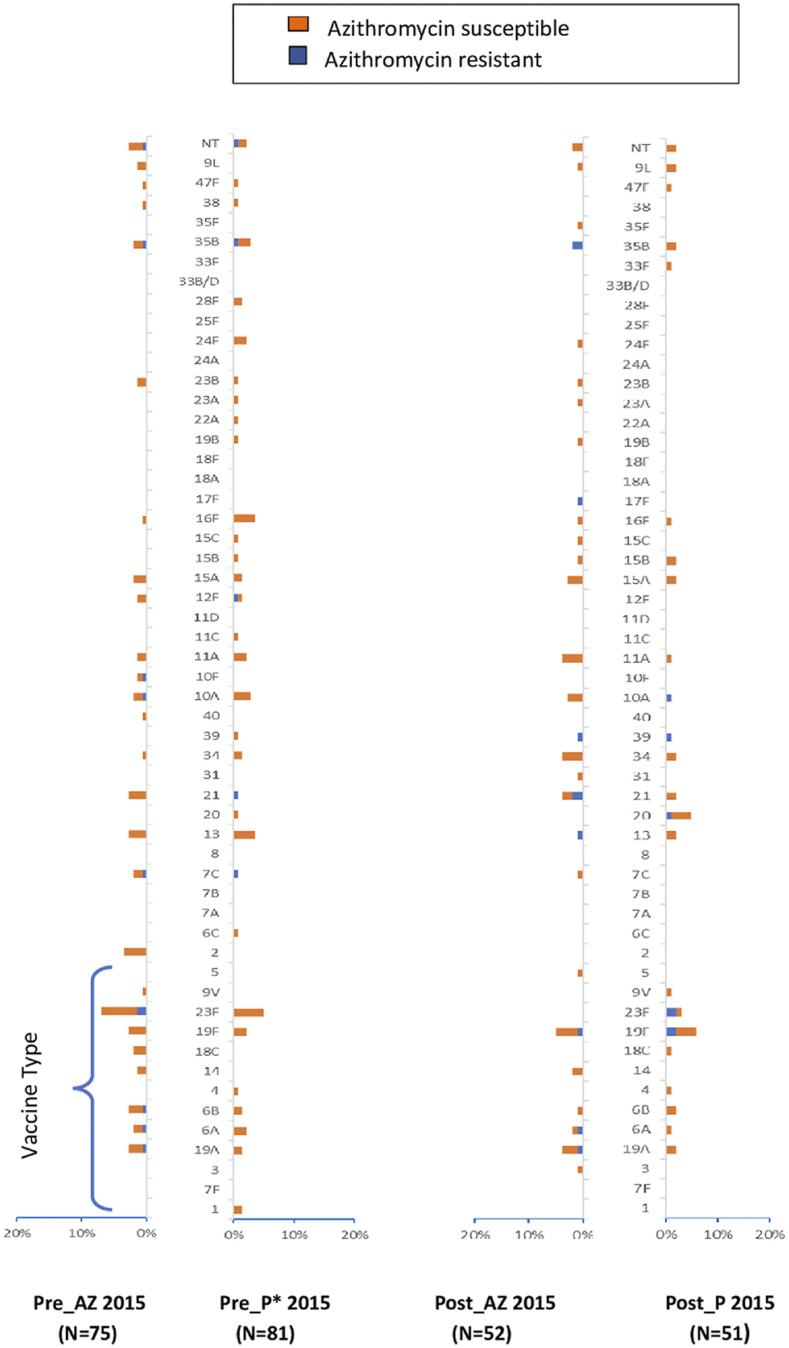
Distribution of nasopharyngeal pneumococcal serotypes before and after azithromycin administration in 2015. The proportions of serotypes detected at the respective study visit are shown: orange bars represent azithromycin-susceptible isolates, and blue bars represent azithromycin-resistant isolates. Pneumococcal conjugate vaccination 13 vaccine-type (VT) serotypes are grouped at the bottom of the figure. This figure appears in color at www.ajtmh.org.

**Figure 4.**
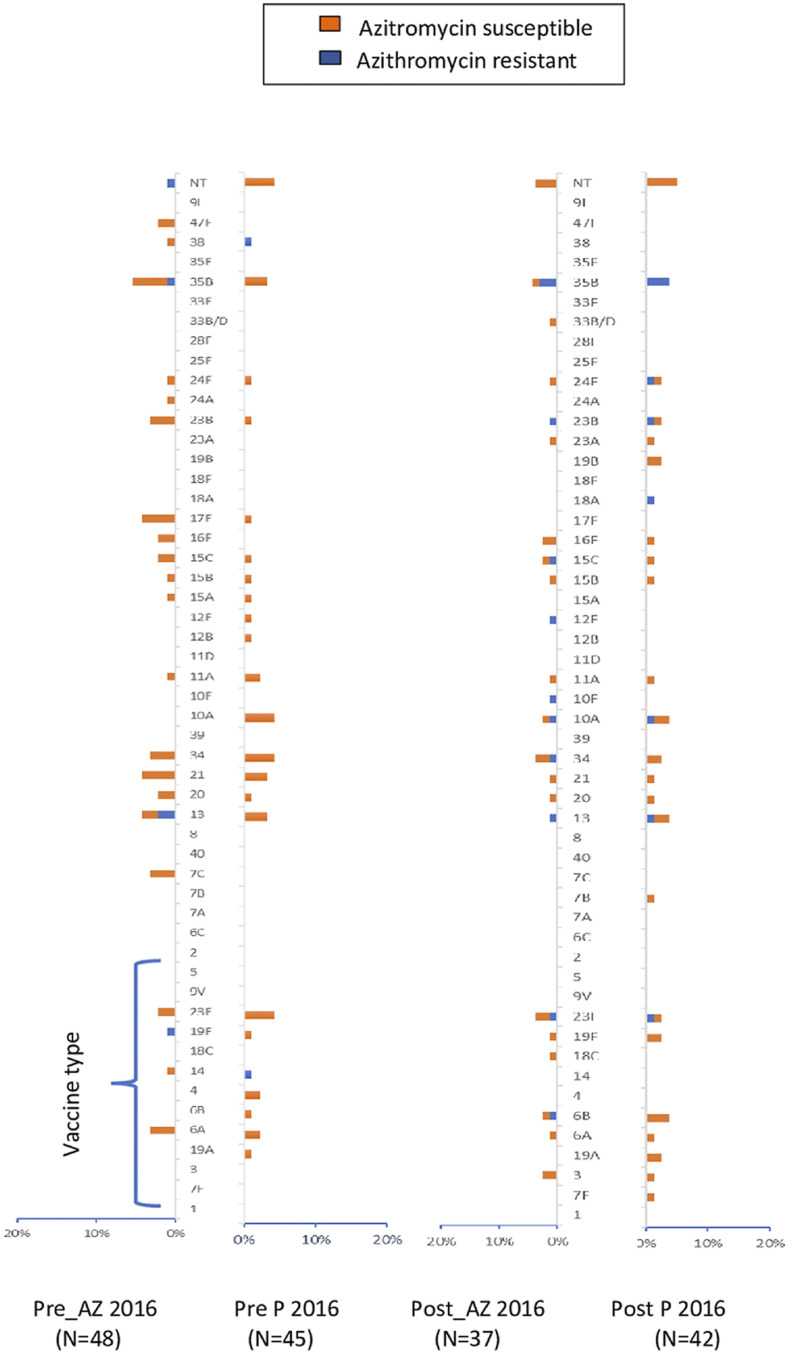
Distribution of nasopharyngeal pneumococcal serotypes before and after azithromycin administration in 2016. The proportions of serotypes detected at the respective study visit are shown: orange bars represent azithromycin-susceptible isolates, and blue bars represent azithromycin-resistant isolates. Pneumococcal conjugate vaccination 13 vaccine-type (VT) serotypes are grouped at the bottom of the figure. This figure appears in color at www.ajtmh.org.

**Figure 5.**
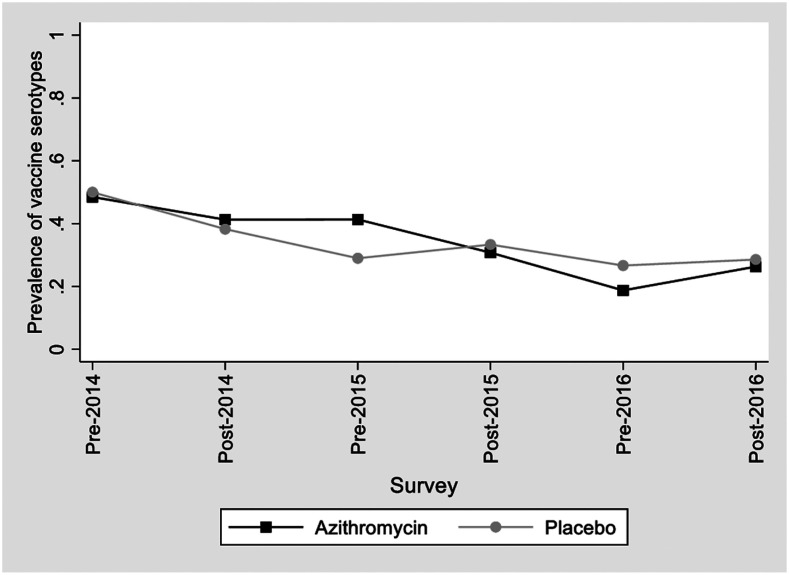
Prevalence of pneumococcal isolates that were of vaccine serotype by treatment arm during the course of the trial.

Nasopharyngeal carriage surveys were carried out before the administration of AZ or placebo in years 2 and 3 of the study. There were substantial differences between the serotype distribution found in the second and third pretreatment surveys compared with the baseline (Figures 2–4), with a significant decrease from baseline in the percentage of VT 49.3–35.4%, *P* = 0.020% and 49.3% versus 22.6%, *P* < 0.001 for years 2015 and 2016, respectively (Figure 5). A change in serotype distribution was noted between the baseline survey and subsequent pre-intervention surveys with some new serotypes appearing, whereas some of those found initially were absent. No significant differences were found in the distribution of serotypes between children in the AZ and placebo group (Figure 5, Supplemental Table S4). The most frequently identified serotypes were 23F, 13, 19F, 35B, and 10A in the 2015 pretreatment survey and 35B, 13, 21, and 34 in the 2016 pretreatment survey.

### Posttreatment surveys.

A diversity of serotypes was seen also in the posttreatment surveys. The predominant serotypes remained very similar to those seen before administration of AZ with 6A, 19F, and 23F predominating (Figures 2–4); 35B was the most predominant serotype among the NVT isolates, with a prevalence ranging from 3.9% to 10.0%. The proportion of vaccine serotypes decreased between the baseline (pretreatment) and the last posttreatment survey (49.3% versus 27.5% *P* = 0.002). This decrease was similar in the two-treatment arm (Figure 5, Supplemental Table S4).

### Serotypes and AZ resistance.

Only three of the 134 children from whom a pneumococcus was isolated in the first pre-intervention baseline survey carried an AZ-resistant strain of *S. pneumoniae* (serotypes 6B, 15A, and 35B). In 2014, the prevalence of AZ-resistant strains of *S. pneumoniae* increased from 2.2% (3/134) in the pretreatment survey to 12.5% (18/144) in the posttreatment survey (*P* = 0.001). This increase involved serotypes 4, 6A, 7F, 14, 15C, 19A, 19F, 21, 23F, 24A, and 35B. In 2015, there was no significant difference between the prevalence of AZ-resistant strains in the pretreatment survey (10.4% [15/144]) versus the posttreatment surveys (16.5% [17/103]), *P* = 0.160.). The serotypes with the highest azithromycin resistance posttreatment were 6A, 19A, 23F, 21, and 35B. However, in 2016, an increase in the prevalence of AZ-resistant strains from 7.5% (7/93) in the pretreatment to 26.6% (21/79) in the posttreatment surveys was observed (*P* = 0.001) (Supplemental Table S4 and Figure S1). Resistant strains included serotypes 6A, 23F, 13, 10A, 15C, 23B, and 35B. Overall, four serotypes 35B (2%), 23F (0.9%), 19F (0.9%), and 6A (0.7%) had the most AZ-resistant isolates.

## DISCUSSION

This study investigated the impact of MDA with AZ given monthly for 4 months each year for 3 years on the distribution of pneumococcal serotypes. During the period of the study, a diversity of serotypes was observed. There was a swift appearance and disappearance of many serotypes between years, but serotypes 6A, 19A, 19F, 23F, and 35B persisted. Similar studies conducted in other African countries observed the same patterns.^4,19^ These prevalent serotypes were found in both pre- and posttreatment and also in children who received or did not receive AZ.

We previously reported that the prevalence of pneumococcal carriage decreased overall during the three years of the study, whereas the prevalence of isolates resistant to AZ increased in both children who received AZ and in children in neighboring households who received placebo, but resistance was more marked in the former.^15^ This increase in resistance of *S. pneumoniae* to AZ, associated with cross-resistance to erythromycin, could have clinical consequences as the latter antibiotic is sometimes used for the treatment of pneumonia and generation of AZ resistance in other bacteria not investigated in this study, for example, gut bacteria, but could also be clinically important.

We considered that the increase in resistance to AZ observed during the course of the study might be due to the emergence of resistance in a single serotype. However, this was not the case as resistance was seen in several serotypes. There was no significant difference in the serotype distribution of children who had received AZ or placebo.

Although administration of AZ did not change the distribution of serotypes, a reduction in carriage with VT was noted as the study progressed. The prevalence of carriage of VT pneumococci decreased from 49.3% in the first baseline survey to 27.5% in the final one. The reduction in VT was seen in both the azithromycin and placebo group (Figure 5) and may have been linked to the introduction of PCV13 into the routine national vaccination program in the year before the study started, with an increasing proportion of children being vaccinated as the study progressed. Not all children had a vaccination card, but based on the information that was available, it is estimated that the proportion of children who had received PCV13 increased from about 64% to 87% during the course of the study.^15^ Carriage of pneumococci declines with age, but the mean age of children in each survey was similar. Carriage with serotypes 1 and 5 was found only infrequently, in four and one child, respectively, as reported in many previous carriage studies carried out in Africa.^4,20–22^ However, pneumococci belonging to these serotypes are important causes of invasive pneumococcal disease in sub-Saharan Africa.

One of the limitations of our study was that because of budgetary constraints, we were only able to serotype approximately 50% of the isolated pneumococcal. However, random sampling should have ensured that the serotype distribution found in different surveys reflected the true situation. In addition, there were limitations in the information on the vaccination status of all children in the study as parents and/or legal guardians were not always able to provide full documentations regarding their child’s vaccination record. An additional weakness of the study was that it was not possible to do whole genome sequencing for financial reasons, and this would have been able to identify if there had been an expansion of a specific clone.

In conclusion, this study has shown that the increase in the resistance of nasopharyngeal isolates of *S. pneumoniae* noted following MDA with AZ used in association with SMC was probably not associated with the emergence of a single resistant clone but due to the emergence of resistance in pneumococci belonging to several different serotypes.

## Supplemental tables and figure

Supplemental materials
