# Supplementary material for: Serotype Profile of Nasopharyngeal Isolates of Streptococcus pneumoniae Obtained from Children in Burkina Faso before and after Mass Administration of Azithromycin
Source: Am J Trop Med Hyg. 2020 Jun 8;103(2):679–83. doi: 10.4269/ajtmh.19-0944 (PMC7410481; doi:10.4269/ajtmh.19-0944)
Supplement: Supplementary file 1 [file tpmd190944.SD1.pdf]

Supplementary Materials

1. Laboratory methods ....p. 2

2. Supplementary analysis...p. 3

3. Supplementary tables ....p. 4

3. Supplementary figures…………………………………………………………………..p. 9

Laboratory Methods

Isolation of Streptococcus pneumoniae and determination of antimicrobial resistance

Following storage of cryotubes containing skimmed milk-tryptone-glucose-glycerol medium (STGG) inoculated with a nasopharyngeal swab at -80°C, 200µl of STGG medium were removed and seeded in Todd Hewitt enrichment medium with 0.5% yeast extract plus 1mL rabbit serum (THY) and incubated for 6 hours in a humid atmosphere containing 5% CO_2 at 37 ^{O}C. After 18 to 24 hours of incubation, 10µl of THY were spread on a gentamicin blood agar plate and incubated for 18-24 hours at 37 ^{O}C in 5% CO_2. Plates were then examined for streptococcal-like alpha-hemolytic colonies. A single alpha hemolytic colony was picked and sub-cultured 24 hours later on a blood agar medium. Pneumococcal identification was based on colony morphology and conventional characterization methods (optochin sensitivity and biliary solubility tests) [1].

The Kirby-Bauer disc diffusion method was used to test pneumococcal isolates for sensitivity to azithromycin (15ug), penicillin (oxacillin 1ug), norfloxacine (10ug) and vancomycin (30ug) according to the recommendations of the Clinical and Laboratory Standards Institute (CLSI)[2]. Azithromycin and penicillin resistance were confirmed by Epsilometer-test (E-test) with strips of azithromycin and penicillin respectively (Oxoid Ltd). The minimum inhibitory concentration (MICs) were determined. The ceftriaxone E-test was also performed to monitor resistance to this antibiotic. Isolates could not be tested against all antibiotics on all occasions because of problems over the supply of reagents but assays for azithromycin and erythromycin were conducted during each survey using standardised methods.

Determination of Streptococcus pneumoniae serotypes by conventional PCR

Nasopharyngeal swab samples that tested positive for *S. pneumoniae* were serotyped using a multiplex PCR assay optimized for African clinical samples. Pneumococcal strains isolated and frozen at -80°C in physiological water underwent a thermal shock by heating in 100°C to release DNA from the bacteria. The primer concentrations and cycling conditions were as recommended by the United

States Centers for Disease Control and Prevention protocol [1] and expanded by the CDC to include up to 39 serotypes divided into 8 reactions. (Table S1)

All pneumococci were screened first with a cpsA PCR amplification and those that were cpsA positive were subsequently analyzed in the sequential PCRs including 5 primers pairs for different serotypes or group of serotypes.

Determination of unique serotypes by Quellung test and agglutination test.

All non-typable serotypes (90) and serotype groups (224) obtained by conventional PCR were sent to the laboratory of the Respiratory Branch Division at the CDC to obtain unique serotypes for serotype groups and for non-typable serotypes to obtain unique serotypes by the standard Neufeld capsular reaction test (Quellung test). This method involves testing a pneumococcal cell suspension with pooled and specific antisera directed against the capsular polysaccharide. The antigen-antibody reactions are observed microscopically. A positive Quellung reaction is the result of the binding of the capsular polysaccharide of pneumococci with type specific antibody contained in the typing antiserum. (3)

References.

1. Da Gloria Carvalho M, Pimenta FC, Jackson D, et al. Revisiting pneumococcal carriage by use of broth enrichment and PCR techniques for enhanced detection of carriage and serotypes. J Clin Microbiol **2010**; 48: 1611-8.
2. Clinical and laboratory Standards Institute. Performance standards for antimicrobial disc susceptibility tests: approved standard, 26th ed. N1006. 2014
3. NCIRD-CDC, *Identification and Characterization of Streptococcus pneumoniae*, in *Laboratory Methods for the Diagnosis of Meningitis*, CDC, Editor. April 15, 2016. p. 14.

SUPPLEMENTARY TABLES

Table S1 : Primers for conventionnal PCR

Primers	Primer Sequence (5' – 3')	Product Size (bp)
1-f	CTC TAT AGA ATG GAG TAT ATA AAC TAT GGT TA	280
1-r	CCA AAG AAA ATA CTA ACA TTA TCA CAA TAT TGG C	
2-f	TAT CCC AGT TCA ATA TTT CTC CAC TAC ACC	290
2-r	ACA CAA AAT ATA GGC AGA GAG AGA CTA CT	
3-f	ATG GTG TGA TTT CTC CTA GAT TGG AAA GTA G	371
3-r	CTT CTC CAA TTG CTT ACC AAG TGC AAT AAC G	
4-f	CTG TTA CTT GTT CTG GAC TCT CGA TAA TTG G	430
4-r	GCC CAC TCC TGT TAA AAT CCT ACC CGC ATT G	
5-f	ATA CCT ACA CAA CTT CTG ATT ATG CCT TTG TG	362
5-r	GCT CGA TAA ACA TAA TCA ATA TTT GAA AAA GTA TG	
6A/6B/6C/6D-f	AAT TTG TAT TTT ATT CAT GCC TAT ATC TGG	250
6A/6B/6C/6D-r	TTA GCG GAG ATA ATT TAA AAT GAT GAC TA	
6C/6D-f	CAT TTT AGT GAA GTT GGC GGT GGA GTT	727
6C/6D-r	AGC TTC GAA GCC CAT ACT CTT CAA TTA	
7C/(7B/40)-f	CTA TCT CAG TCA TCT ATT GTT AAA GTT TAC GAC GGG A	260
7C/(7B/40)-r	GAA CAT AGA TGT TGA GAC ATC TTT TGT AAT TTC	
7F/7A-f	TCC AAA CTA TTA CAG TGG GAA TTA CGG	599
7F/7A-r	ATA GGA ATT GAG ATT GCC AAA GCG AC	
8-f	GAA GAA ACG AAA CTG TCA GAG CAT TTA CAT	201
8-r	CTA TAG ATA CTA GTA GAG CTG TTC TAG TCT	
9N/9L-f	GAA CTG AAT AAG TCA GAT TTA ATC AGC	516
9N/9L-r	ACC AAG ATC TGA CGG GCT AAT CAA T	
9V/9A-f	GGG TTC AAA G TC AGA CAG TG A ATC TTA A	816
9V/9A-r	CCA TGA ATG A AA TCA ACA TT G TCA GTA GC	
10A- f	GGT GTA GAT TTA CCA TTA GTG TCG GCA GAC	628
10A-r	GAA TTT CTT CTT TAA GAT TCG GAT ATT TCT C	
10F/(10C/33C)- f	GGA GTT TAT CGG TAG TGC TCA TTT TAG CA	248
10F/(10C/33C)-r	CTA ACA AAT TCG CAA CAC GAG GCA ACA	
11A/11D-f	GGA CAT GTT CAG GTG ATT TCC CAA TAT AGT G	463
11A/11D-r	GAT TAT GAG TGT AAT TTA TTC CAA CTT CTC CC	
12F/(12A/44/46)-f	GCA ACA AAC GGC GTG AAA GTA GTT G	376
12F/(12A/44/46)-r	CAA GAT GAA TAT CAC TAC CAA TAA CAA AAC	
13-f	TAC TAA GGT AAT CTC TGG AAA TCG AAA GG	655
13-r	CTC ATG CAT TTT ATT AAC CG C TTT TTG TTC	
14-f	GAA ATG TTA CTT GGC GCA GGT GTC AGA ATT	189
14-r	GCC AAT ACT TCT TAG TCT CTC AGA TGA AT	
15A/15F-f	ATT AGT ACA GCT GCT GGA ATA TCT CTT C	434
15A/15F-r	GAT CTA GTG AAC GTA CTA TTC CAA AC	
15B/15C-f	TTG GAA TTT TTT AAT TAG TGG CTT ACC TA	496
15B/15C-r	CAT CCG CTT ATT AAT TGA AGT AAT CTG AAC C	
16F-f,	GAA TTT TTC AGG CGT GGG TGT TAA AAG	717
16F-r	CAG CAT ATA GCA CCG CTA AGC AAA TA	
17F-f	TTC GTG ATG ATA ATT CCA ATG ATC AAA CAA GAG	693
17F-r	GAT GTA ACA AAT TTG TAG CGA CTA AGG TCT GC	
18/(18A/18B/18C/18F)-f	CTT AAT AGC TCT CAT TAT TCT TTT TTT AAG CC	573
18/(18A/18B/18C/18F)-r	TTA TCT GTA AAC CAT ATC AGC ATC TGA AAC	
19A-f	GAG AGA TTC ATA ATC TTG CAC TTA GCC A	566
19A-r	CAT AAT AGC TAC AAA TGA CTC ATC GCC	
19F-f	GTT AAG ATT GCT GAT CGA TTA ATT GAT ATC C	304
19F-r	GTA ATA TGT CTT TAG GGC GTT TAT GGC GAT AG	
19Fvar-f	GAC AAT TCT GGT TGA CTT GTT GAT TTT G	585
19Fvar-r	CTA CCA AAT ACC TCA CCA GCT TCC	
20-f	GAG CAA GAG TTT TTC ACC TGA CAG CGA GAA G	514
20-r	CTA AAT TCC TGT AAT TTA GCT AAA ACT CTT ATC	
21-f	CTA TGG TTA TTT CAA CTC AAT CGT CAC C	192

21-r	GGC AAA CTC AGA CAT AGT ATA GCA TAG	
22F/22A-f	GAG TAT AGC CAG ATT ATG GCA GTT TTA TTG TC	643
22F/22A-r	CTC CAG CAC TTG CGC TGG AAA CAA CAG ACA AC	
23A-f	TAT TCT AGC AAG TGA CGA AGA TGC G	722
23A-r	CCA ACA TGC TTA AAA ACG CTG CTT TAC	
23B-f	CCA CAA TTA G CG CTA TAT TCA TTC AAT CG	199
23B-r	GTC CAC GCT GAA TAA AAT GAA GCT CCG	
23F-f *a*	GTA ACA GTT GCT GTA GAG GGA ATT GGC TTT TC	384
23F-r	CAC AAC ACC TAA CAC TCG ATG GCT ATA TGA TTC	
24/(24A, 24B, 24F)-f	GCT CCC TGC TAT TGT AAT CTT TAA AGA G	99
24/(24A, 24B, 24F)-r	GTG TCT TTT ATT GAC TTT ATC ATA GGT CGG	
31-f	GGA AGT TTT CAA GGA TAT GAT AGT GGT GGT GC	701
31-r	CCG AAT AAT ATA TTC AAT ATA TTC CTA CTC	
33F/(33A/37)-f	GAA GGC AAT CAA TGT GAT TGT GTC GCG	338
33F/(33A/37)-r	CTT CAA AAT GAA GAT TAT AGT ACC CTT CTA C	
34-f	GCT TTT GTA AGA GGA GAT TAT TTT CAC CCA AC	408
34-r	CAA TCC GAC TAA GTC TTC AGT AAA AAA CTT TAC	
35A/(35C/42)-f	ATT ACG ACT CCT TAT GTG ACG CGC ATA	280
35A/(35C/42)-r	CCA ATC CCA AGA TAT ATG CAA CTA GGT T	
35B-f	GAT AAG TCT GTT GTG GAG ACT TAA AAA GAA TG	677
35B-r	CTT TCC AGA TAA TTA CAG GTA TTC CTG AAG CAA G	
35F/47F-f	GAA CAT AGT CGC TAT TGT ATT TTA TTT AAA GCA A	517
35F/47F-r	GAC TAG GAG CAT TAT TCC TAG AGC GAG TAA ACC	
38/25F/25A-f	CGT TCT TTT ATC TCA CTG TAT AGT ATC TTT ATG	574
38/25F/25A-r	ATG TTT GAA TTA AAG CTA ACG TAA CAA TCC	
39-f	TCA TTG TAT TAA CCC TAT GCT TTA TTG GTG	98
39-r	GAG TAT CTC CAT TGT ATT GAA ATC TAC CAA	
*cps*A-f	GCA GTA CAG CAG TTT GTT GGA CTG ACC	160
*cps*A-r	GAA TAT TTT CAT TAT CAG TCC CAG TC	

Table S2: Algorithm of conventional multiplex PCR suitable for African isolates

	Primers	Amplified serotypes
**Reaction 1**		
	14	14
	1	1
	5	5
	4	4
	18	18/C/18F/18B/18A-F
**Reaction 2**		
	6	6A/6B/6C/6D
	19	19F
	23	23F
	25	25F/25A/38
	9	9V/9A
**Reaction 3**		
	7	7C/7B/40
	3	3F
	15	15B/15C
	7	7F/7A
	17	17F
**Reaction 4**		
	8	8
	12	12F/12A/44/46
	9	9N/9L
	22	22F/22A
	23	23A
**Reaction 5**		
	24	24F/24A/24B
	2	2
	11	11A/11D
	19A	19A
	16	16F
**Reaction 6**		
	21	21
	33	33F/33A/37
	15	15A/15F
	35	35F/47F
	13	13
**Reaction 7**		
	39	39
	23B	23B
	35	35A/35C/42
	20	20
	35	35B
**Reaction 8**		
	10	10F/10C/33C
	34	24
	10	10A
	31	31

Table S3: Proportions of VT and NVT of serotypes

	Surveys											
	**Pre 2014 (N=430)**	**Post 2014 (N=418)**		**Pre 2015 (N=401)**		**Post 2015 (N=388)**		**Pre 2016 (N=385)**		**Post 2016 (N=396)**		**TOTAL**
**Samples serotyped (n)**	134	144		144		103		93		80		698 (100%)
**VT proportion n (%)**	66 (49.25%)	57 (39.58%)		51 (35.42%)		33 (32.04%)		21 (22.58%)		22 (27.5%)		250 (35.82)
**By Arm**	**NA**	**SMC+P**	**SMC+AZ**	**SMC+P**	**SMC+AZ**	**SMC+P**	**SMC+AZ**	**SMC+P**	**SMC+AZ**	**SMC+P**	**SMC+AZ**	
**VT n (%)**	66 (49.25)	31(38.27)	26(41.27)	20(28.99)	31(41.33)	17(33.33)	16(30.77)	12(26.67)	9(18.75)	12(28.57)	10(26.32)	250 (35.82)
**Proportion Ratio (95% CI)**	0.50 (0.41,0.58)	0.39 (0.32, 0.48)		0,35 (0.28, 0.44)		0.32 (0.24, 0.42)		0.23 (0.15, 0.32)		0.27 (0.19, 0.38)		NA
**NVT n (%)**	68 (50.75)	50 (61.73)	37 (58.73)	49 (71.01)	44 (58.67)	34 (66.67)	36 (69.23)	33 (73.33)	39 (81.25)	30 (71.43)	28 (73.68)	448 (64.18)
**Proportion Ratio (95% CI)**	0.51 (0.42, 0.59)	0.60 (0.52, 0.68)		0.65 (0.56, 0.72)		0.68 (0.58, 0.76)		0.77 (0.68, 0.83)		0.73 (0.62, 0.81)		NA

Table S4. Comparison of the prevalence of vaccine serotypes and non-vaccine serotypes between azithromycin and placebo by survey.

		Crude PR	$CI_{95\%}$	p-value
		**Vaccine serotypes**		
Pre-2014	Azithromycin	0.97	[0.60-1.57]	0.901
	Placebo	1	---	
Post-2014	Azithromycin	1.08	[0.64-1.82]	0.777
	Placebo	1	---	
Pre-2015	Azithromycin	1.43	[0.81-2.50]	0.216
	Placebo	1	---	
Post-2015	Azithromycin	0.92	[0.47-1.82]	0.818
	Placebo	1	---	
Pre-2016	Azithromycin	0.70	[0.30-1.67]	0.424
	Placebo	1	---	
Post-2016	Azithromycin	0.92	[0.40-2.13]	0.848
	Placebo	1	---	

SUPPLEMENTARY FIGURE

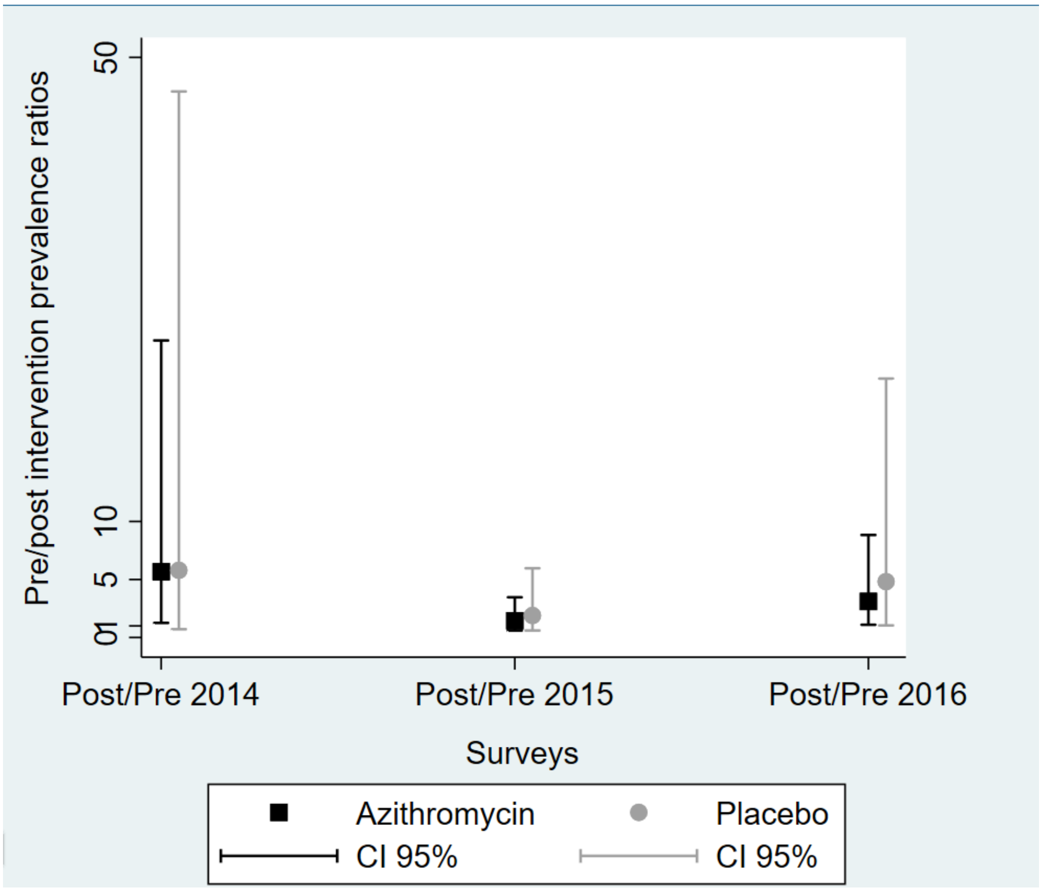

Figure S1: Prevalence ratios of azithromycin resistance between surveys by year and arm
